# A Smart Nanoprobe for Visually Investigating the Activation Effect of Cyclical DOX Release on the p53 Pathway and Pathway-Related Molecules

**DOI:** 10.3390/bios15060383

**Published:** 2025-06-13

**Authors:** Ping Sun, Chunlei Gao, Zhe Chen, Siyu Wang, Gang Li, Mingming Luan, Yaoguang Wang

**Affiliations:** 1 School of Chemistry and Chemical Engineering, Qilu University of Technology (Shandong Academy of Sciences), Jinan 250353, China; 17861405949@163.com (P.S.); gcl02060506@163.com (C.G.); 17861405811@163.com (Z.C.); 13188743389@163.com (S.W.); 2 Shanghai Wanhou Biotechnology Co., Ltd., Shanghai 201815, China; lee@shwanhou.com

**Keywords:** p53 pathway activation, miRNA-34a, apoptosis, visual imaging, fluorescent nanoprobe

## Abstract

Developing appropriate methods for real-time in situ investigation of how drugs influence signaling pathways and related biomolecules holds enormous potential for helping to provide an understanding of how anticancer drugs exert their effects. Herein, we report a smart nanoprobe, PDA-MB (DOX)-Pep, constructed on the basis of polydopamine nanoparticles (PDA NPs) modified with a dense shell of molecular beacon (MB) with embedded doxorubicin (DOX) and peptide, which can respond specifically to miRNA-34a and Caspase-3 targets. Intracellular experiments demonstrated that, in comparison to the control nanoprobe PDA-MB-Pep, the smart nanoprobe could selectively respond to miRNA-34a, facilitating the release of the embedded DOX. The released DOX subsequently activated the p53 pathway, which further upregulated miRNA-34a expression, leading to additional DOX release. This initiated a cyclical process involving the probe’s response to miRNA-34a, DOX release, p53 activation, and miRNA-34a upregulation, ultimately enhancing cell apoptosis and increasing Caspase-3 expression. The designed smart nanoprobe offers a visual approach to explore how anticancer drugs influence signaling pathways and related molecules at the cellular level.

## 1. Introduction

Cancer is one of the most devastating diseases globally, and poses a significant global health challenge. Chemotherapy, as one of the typical therapeutic treatments, is often limited by drug resistance and cancer recurrence [1,2,3,4]. Studies have demonstrated that chemotherapeutic drugs can induce apoptosis by influencing signaling pathways and related biomolecules [5,6,7,8,9]. Extensive studies have revealed that miRNAs play significant roles in the initiation and progression of cancer [10,11,12,13]. For example, miRNA-34a has lower expression in many human cancers, and has been frequently thought to be a tumor suppressor. Combination therapy involving miRNA-34a and doxorubicin (DOX) has been shown to synergistically inhibit the progression of DOX-resistant breast cancer [14,15]. Recently, it was shown that the overexpression of miRNA-34a induced by p53 can enhance the sensitivity of cells to chemotherapy and lead to cell apoptosis [16,17]. As the guardian of the genome, p53 plays a role in tumor inhibition through multiple transcriptional target genes. p53, which is located at a nexus of cellular pathways, is activated in response to cellular stress, DNA damage, and abnormal mitogenic stimulation, leading to the promotion of apoptosis, DNA repair, and growth arrest [18,19,20]. Previous reports have indicated that DNA-damage-inducing agents such as DOX activate p53, which subsequently triggers the expression of many apoptosis-related genes, including miRNA-34a [21]. Hussein’s group found that downregulation of miRNA-34a expression in cisplatin-induced nephrotoxicity in rats was mediated by p53 [22]. In addition, p53 was demonstrated to be an essential mediator for the induction of miRNA-34a in HCT116 cells after DNA damage caused by DOX drugs, which contributed to the promotion of cell apoptosis [23]. Thus, revealing the effects of such drugs on signaling pathways and the critical biomolecules in these pathways is of great significance for improving the precise treatment of cancer.

**Scheme 1:**
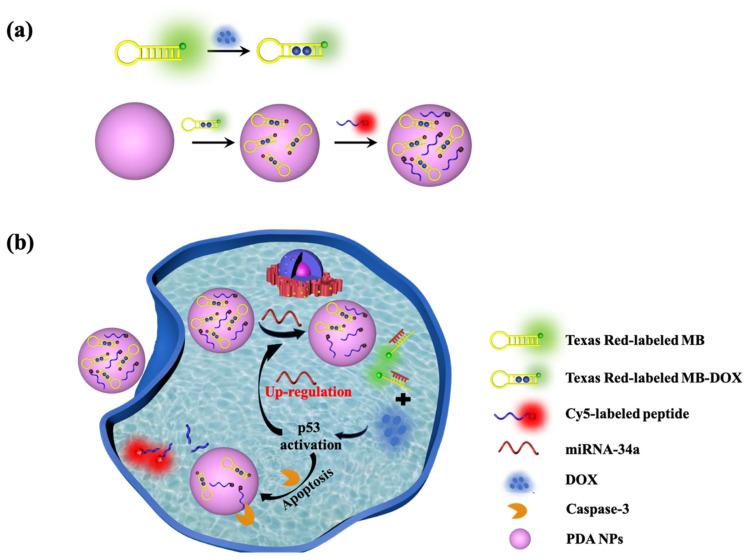
(**a**) Preparation of the smart nanoprobe PDA-MB(DOX)-Pep; (**b**) a schematic illustration of the smart nanoprobe PDA-MB(DOX)-Pep used to visualize the impact of cyclical DOX release on the activation of the p53 pathway and pathway-related miRNA-34a and Caspase-3.

At present, the most commonly applied techniques for studying the effect of drugs on signaling pathways and key biomolecules in pathways are biological methods, including real-time reverse-transcription polymerase chain reaction (RT-PCR) and Western blot (WB), which often require cell lysis and are cumbersome to manage [24,25,26,27]. Consequently, reliable techniques for real-time in situ visualization of the changes in critical molecules in pathways are urgently needed in order to evaluate how anticancer drugs influence signaling pathways. Recently, fluorescent imaging analysis based on nanomaterials has garnered considerable attention, due to its advantages of non-destructivity, high selectivity, and visualization [28,29,30,31,32]. Some nanoparticles have been employed in signal transduction or drug activation pathways. For instance, Tang’s group developed a CuS NP-based switch for photothermal activation of TRPV1 signaling to attenuate atherosclerosis in vascular smooth muscle cells (VSMCs) using near-infrared light [33]. Similarly, Gao et al. fabricated a multifunctional nanoplatform AuNP flares@MSN (S-crizotinib). Upon MTH1 mRNA binding, the entrapped MTH1 inhibitor S-crizotinib was released, which has significant cytotoxicity to cancer cells [34]. Furthermore, Gao’s team developed multifunctional Au–ZnO hybrid NPs for the targeted induction of lysosomal membrane permeabilization (LMP)-dependent apoptosis in cancer cells and real-time imaging [35]. In this regard, a novel smart nanoprobe, PDA-MB(DOX)-Pep, was designed for visually investigating DOX-induced activation of the p53 pathway, as well as the impacts of p53 activation on miRNA-34a and Caspase-3. The molecular beacon (MB) containing GC bases can specifically bind to DOX and quench the fluorescence of DOX [36,37]. Subsequently, the molecular beacon (doxorubicin) (MB(DOX)) and peptide were gradually integrated into the surface of polydopamine nanoparticles (PDA NPs) via electrostatic interactions and π-π stacking [38,39]. Typically, the fluorescence of both Texas Red and Cy5 labeled on the MB and peptide was quenched due to the FRET effect between the PDA NPs and the two dyes [40,41]. When miRNA-34a targets were present, the targets specifically triggered the unwinding of MB by generating duplexes, causing the release of DOX and fluorescence recovery of Texas Red [42,43]. DOX release could activate the p53 signaling pathway, and p53 activation further upregulated miRNA-34a expression, thereby creating a cyclical process of DOX release, p53 activation, and miRNA-34a upregulation, which finally provoked apoptosis. Upon encountering Caspase-3, the peptide underwent specific cleavage, thereby detaching the Cy5 fluorophore from PDA NPs and recovering the fluorescence. Scheme 1 illustrates the specifics of this approach.

## 2. Materials and Methods

### 2.1. Synthesis of PDA NPs

PDA NPs were synthesized according to the method reported in [44]. Dopamine hydrochloride (100 mg) was dissolved in a mixture of Tris/ethanol (V:V = 5:1) and stirred for 24 h. Subsequently, the PDA NPs were centrifuged (10 min, 10,000 rpm) and washed three times with ultrapure water. The preparation process was conducted in the dark.

### 2.2. Fluorescence Quenching Assay

For the purpose of acquiring PDA-MB (DOX) NPs, a Texas Red-labeled MB (DOX) (500 nM) was added to various concentrations of PDA NPs (0–50 μg/mL, 1 mM CaCl_2_, pH 7.4) and shaken for 1 h. Afterward, the fluorescence signal from the Texas Red was first detected at λex/λem = 595 nm/615 nm, respectively, and the most favorable amount of PDA NPs was identified according to a quenching experiment. Next, Cy5-labeled peptide (1 μM) was added to varying concentrations of PDA-MB (DOX) NPs (0–90 μg/mL, 1 mM CaCl_2_, pH 7.4) and shaken for 2 h to obtain PDA-MB (DOX)-Pep. The fluorescence intensity at 660 nm was collected using excitation light at 625 nm to determine the optimal amount of PDA-MB(DOX) NPs.

### 2.3. Preparation of the PDA-MB (DOX)-Pep Nanoprobe

Based on the results of the experiment described above, the MB solution (500 nM) was combined with DOX (50 μM) and shaken at room temperature for 1 h. Subsequently, the PDA NPs (70 μg/mL) were incubated with the above solution and further stirred for 0.5 h, followed by the addition of peptide (1 μM) for 2 h. Finally, the resulting PDA-MB (DOX)-Pep nanoprobe was purified by centrifugation and stored in PBS (pH 7.4) at 4 °C. The preparation of PDA-MB-Pep as a control probe was similar to that for PDA-MB (DOX)-Pep, except that DOX was not added.

### 2.4. Targeted Regulation of DOX Release by miRNA-34a

To study DOX release from the PDA-MB (DOX)-Pep nanoprobe, the fluorescence changes of DOX were measured upon addition of an miRNA-34a target strand to nanoprobe. MiRNA-34a (5 μM) was introduced to the probe solution (70 μg/mL) and incubated for 4 h at 37 °C. The fluorescence intensity of DOX at 560 nm was measured at an excitation wavelength of 490 nm. The experiment was conducted in three parallel groups.

### 2.5. Fluorescence Response of PDA-MB (DOX)-Pep Nanoprobe

To detect the response of miRNA-34a to the nanoprobe, varying concentrations of miRNA-34a target strands (0–240 μM) were added to the probe solution (70 μg/mL), followed by incubation at 37 °C for 2 h. The fluorescence signal of Texas Red was then recorded at λex/λem = 595 nm/615 nm. Similarly, different concentrations of Caspase-3 (0–4 U) were added to the probe solution and incubated at 37 °C for 4 h. The fluorescence intensity of Cy5 was recorded at λex/λem = 625 nm/660 nm.

### 2.6. Confocal Fluorescence Imaging

To investigate the visualization of the nanoprobe for studying drug activation of the p53 pathway and its impact on miRNA and apoptosis, HeLa and HepG-2 cells were selected and divided into two parallel groups. One group was treated with 7 μg/mL PDA-MB (DOX)-Pep, while the other group was treated with the control probe PDA-MB-Pep at a concentration of 7 μg/mL. After being incubated for different times (0–72 h), fluorescence imaging of the cells was performed using a confocal laser scanning microscope (CLSM) (detailed parameters: frame size: 1024 × 1024, speed: 600 Hz, bidirectional X, image size: 184.52 μm × 184.52 μm, zoom: 1.00, and smart gain: 800 V). In order to observe the release of DOX, the fluorescence signals of DOX (500–580 nm) were collected at an excitation wavelength of 490 nm. Furthermore, the excitation wavelengths used for Cy5 and Texas Red were 625 nm and 525 nm, with corresponding emission ranges of 600–620 nm and 630–710 nm, respectively.

### 2.7. Apoptosis Detection

Cell apoptosis was evaluated using an apoptosis kit after incubation with the nanoprobe. HeLa and HepG-2 cells were divided into two parallel groups, and then cultured with 7 μg/mL PDA-MB (DOX)-Pep and PDA-MB-Pep for 48 h. Subsequently, after being stained according to the kit instructions, the cells were examined by CLSM. The excitation wavelengths of GreenNuc™ and Annexin V-mCherry were 500 nm and 587 nm, respectively, corresponding to the collection windows of 510–580 nm and 595–680 nm.

The cell apoptosis induced by the PDA-MB (DOX)-Pep nanoprobe was further studied by Calcein AM/PI double-staining. HeLa and HepG-2 cells were first incubated with 7 μg/mL of the nanoprobe for 0 h, 12 h, and 48 h, respectively. Next, a living/dead cell double-staining test was employed according to the kit instructions, and the staining results were detected by CLSM. The fluorescence signals of Calcein AM and PI were measured at λex/λem = 494/517 nm and λex/λem = 535/617 nm.

## 3. Results and Discussion

### 3.1. Interaction Between MB and DOX

According to a previous report, the fluorescence of DOX can be quenched upon interaction with GC base pairs [45]. To determine the DOX-loading capacity, DOX fluorescence was measured after incubating DOX with varying concentrations of MB. Progressive decreases in the fluorescence intensity of DOX (1 μM) were observed with an increase in MB concentration up to 500 nM, which indicated maximal fluorescence quenching (Figure S1a,b). The results showed that two DOX molecules could intercalate into a single MB in line with the designed DNA sequences (Table S1). The UV-vis spectrum (Figure S1c) shows that the MB (DOX) complex has characteristic absorption peaks for DOX and the MB at 480 nm and 595 nm, confirming the successful preparation of the MB (DOX).

### 3.2. Synthesis and Characterization

**Figure 1:**
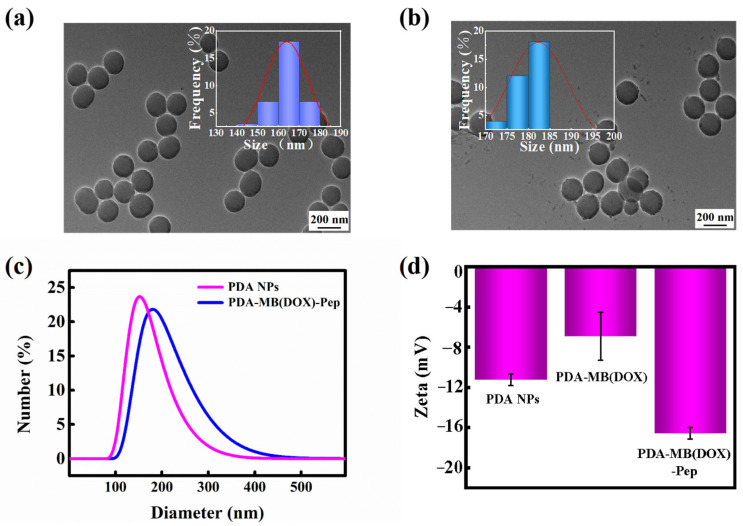
(**a**) TEM images of PDA NPs; inset: size distribution of PDA NPs. (**b**) TEM images of PDA-MB(DOX)-Pep; inset: size distribution of PDA-MB(DOX)-Pep (scale bar: 200 nm). (**c**) DLS analysis of PDA NPs and PDA-MB(DOX)-Pep. (**d**) Zeta potential of PDA NPs, PDA-MB(DOX), and PDA-MB(DOX)-Pep.

For the next step, PDA NPs were prepared by self-polymerization of DA•HCl. As shown in Figure S2, the FTIR spectrum of the PDA NPs displays characteristic peaks at 1635 cm^−1^ (C=C stretching) and at 3220 cm^−1^ (O-H and N-H stretching), which are consistent with previous studies [46,47,48]. The morphology and particle size distribution of the PDA NPs, determined by TEM analysis, are shown in Figure 1a. The TEM images reveal that the PDA NPs had uniform spherical shapes with a diameter of about 162 nm and good dispersion. After modification with the MB (DOX) and peptide, the nanoprobe size increased to approximately 185 nm. The DLS (Figure 1c) and SEM (Figure S3a,b) results further verify the TEM results. EDS analysis (Figure S3c,d) indicated that the nanoprobe contained not only the elements C, N, and O, but also Cl, S, and P, in contrast to the PDA NPs, showing that the peptide and MB (DOX) were successfully loaded onto the PDA NPs. Zeta-potential experiments (Figure 1d) confirmed the successful preparation of the nanoprobe in different stages, i.e., PDA NPs (−11.2 mV), PDA-MB (DOX) (−6.9 mV), and PDA-MB(DOX)-Pep (−16.6 mV). The UV-vis absorption spectrum further verifies the results. Figure S4 illustrates that PDA-MB(DOX)-Pep exhibits four typical optical absorption peaks at 260, 480, 595, and 650 nm, in contrast to the PDA NPs; these are attributed to the distinctive absorption peaks associated with MB, DOX, Texas Red, and Cy5, respectively.

To improve the detection performance, the optimal amount of PDA NPs for use as a nanoprobe carrier was estimated by fluorescence quenching. As shown in Figure S5a,b, the fluorescence of the Texas Red labeled-MB (DOX) was gradually extinguished with an increase in the concentration of PDA NPs, until efficient quenching was obtained at a PDA NP concentration of 40 μg/mL. Subsequently, quenching of the Cy5 fluorescence was performed after the Cy5 labeled-peptide solution had been added to different levels of PDA-MB (DOX), indicating that the concentration of 70 μg/mL PDA-MB (DOX) completely quenched the Cy5 fluorescence (Figure S5c,d). Thus, a PDA NP concentration of 70 μg/mL was finally chosen for the subsequent experiment.

### 3.3. In Vitro Investigations of the PDA-MB (DOX)-Pep Nanoprobe

**Figure 2:**
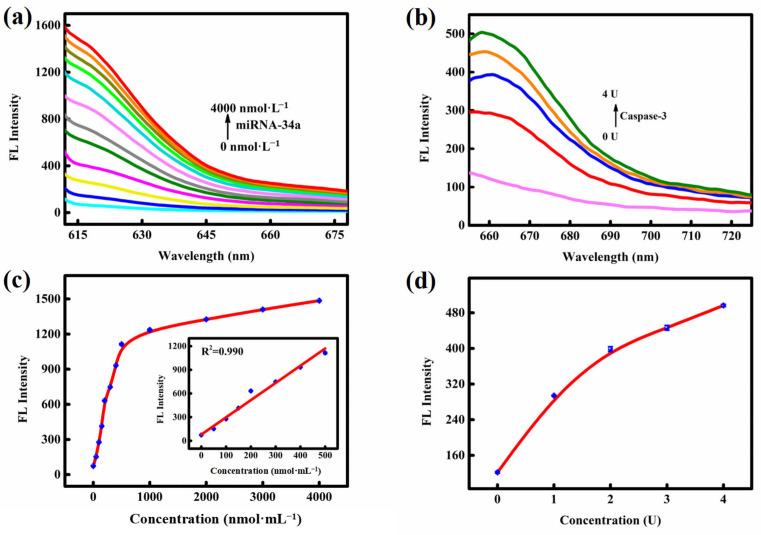
The fluorescence spectra (**a**) and fluorescence intensity (**c**) of the nanoprobe incubated with different concentrations of miRNA-34a (0–4000 nM) for 2 h. Inset is the calibration curve of the Texas Red. The fluorescence spectra (**b**) and fluorescence intensity (**d**) of the nanoprobe treated with different concentrations of Caspase-3 (0–4 U) for 4 h.

**Figure 3:**
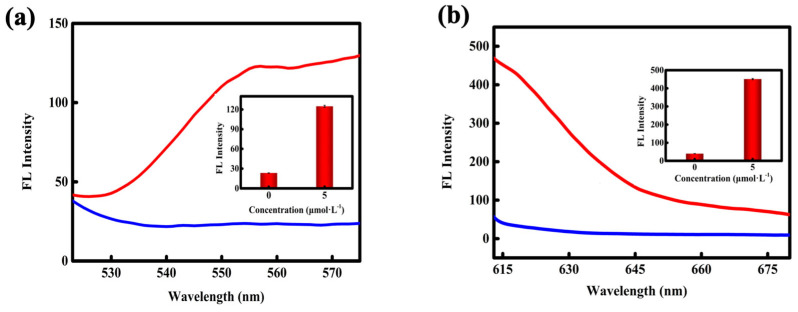
Fluorescence spectra of DOX (**a**) and Texas Red (**b**) before (blue line) and after (red line) addition of miRNA-34a to nanoprobe (inset: fluorescence intensities of DOX at 560 nm and Texas Red at 615 nm).

The performance of the nanoprobe for recognizing miRNA-34a and Caspase-3 targets was further studied. With an increase in the concentrations of the two targets, the fluorescence intensities of Texas Red and Cy5 increased gradually (Figure 2), thus indicating that the nanoprobe could respond to the corresponding target, leading to fluorescence recovery. Moreover, the release of DOX was investigated by incubating the nanoprobe with a sufficient amount of the miRNA-34a target. As shown in Figure 3, the fluorescence signals of DOX and Texas Red were significantly enhanced in the presence of miRNA-34a, while the fluorescence intensities were weak in the absence of the target. The results show that the DOX embedded in the MB could be effectively released when the PDA-MB (DOX)-Pep nanoprobe responded to miRNA-34a.

To study the selectivity of the PDA-MB (DOX)-Pep probe towards Caspase-3 and miRNA-34a, the probe solution was subjected to incubation with the two targets and other interfering substances (miRNA-21, miRNA-221, NaCl, BSA, etc.), respectively. As shown in Figure S6, the nanoprobe only exhibited strong fluorescence responses to the two specific targets. However, there were no obvious fluorescent recoveries of the two dyes with treatment with the other interfering substances, further indicating that the PDA-MB (DOX)-Pep nanoprobe possesses notable specificity. This dynamic study indicated that the nanoprobe was able to respond to the two targets within 3 h (Figure S7).

Subsequently, the stability of PDA-MB (DOX)-Pep was also confirmed. As shown in Figure S8, the fluorescence signals of Texas Red, DOX, and Cy5 remained largely unchanged with solutions of varying pH. Furthermore, the changes over time in the fluorescence intensities of the three substances in cell culture medium were very small. This demonstrates that PDA-MB (DOX)-Pep has significant stability.

### 3.4. MTT Assay

To assess the cytotoxicity of the PDA NPs and the PDA-MB (DOX)-Pep nanoprobe, an MTT assay was performed in HeLa and HepG-2 cells. As shown in Figure S9, the PDA NPs (70 μg/mL) had good biocompatibility, with 72 h survival rates of over 90%. Conversely, cells treated with the nanoprobe (7 μg/mL) exhibited a decrease in survival rate with the extension of incubation time, and the cell survival rate was only 50% after 72 h of incubation, which suggests that the nanoprobe can release DOX and induce cell apoptosis.

### 3.5. Intracellular Study of PDA-MB (DOX)-Pep

**Figure 4:**
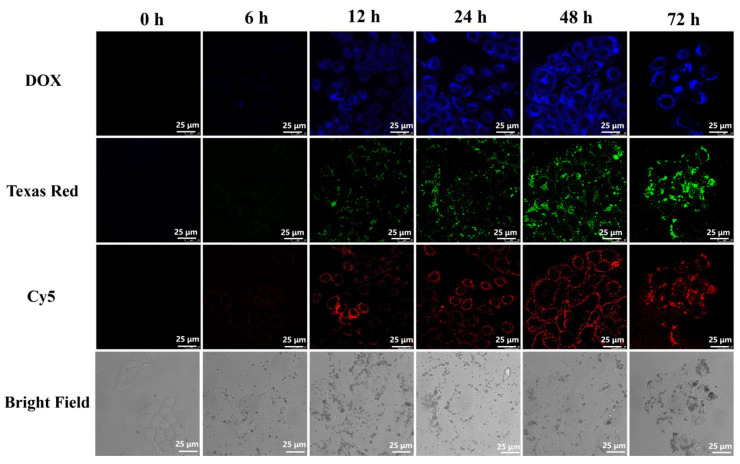
CLSM images of HeLa cells cultured with 7 μg/mL PDA-MB (DOX)-Pep nanoprobe for different durations. The three channels were monitored at excitation wavelengths of 490, 595, and 625 nm, from top to bottom. Scale bars: 25 μm.

**Figure 5:**
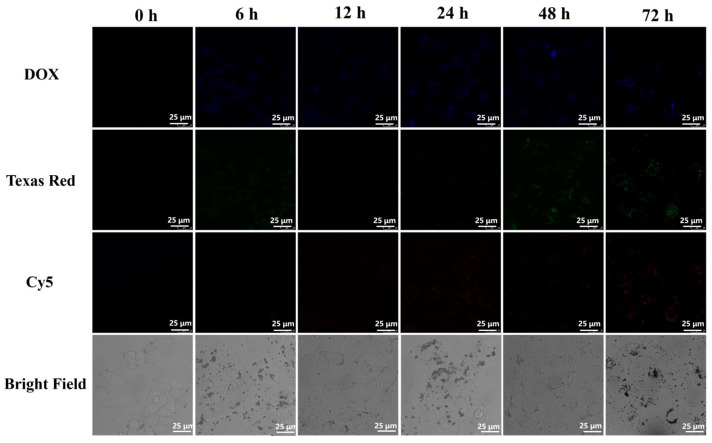
CLSM images of HeLa cells cultured with 7 μg/mL PDA-MB-Pep nanoprobe for different durations. The three channels were monitored at excitation wavelengths of 490, 595, and 625 nm, from top to bottom. Scale bars: 25 μm.

Previous studies have demonstrated that DNA-damage-inducing agents may activate the p53 pathway, which, in turn, initiates the expression of various apoptosis-related genes, including miRNA-34a [22]. To explore the feasibility of the nanoprobe for visually investigating drug-induced activation of the p53 pathway, as well as the effects of p53 activation on miRNA-34a and Caspase-3, HeLa cells were first chosen and divided into two groups. The two groups of cells were treated with the PDA-MB (DOX)-Pep smart probe and the control probe PDA-MB-Pep, respectively. After incubation for different durations, the blue fluorescence signals of DOX, the green fluorescence of Texas Red for miRNA-34a, and the red fluorescence of Cy5 for Caspase-3, respectively, were monitored with CLSM (Figure 4 and Figure 5). The CLSM imaging shows that the three fluorescence signals in cells incubated with the two probes were notably weak at 0 and 6 h, suggesting that miRNA-34a expression was minimal in the cancer cells, and both DOX release and apoptosis were infrequent. Notably, in cells treated with the PDA-MB (DOX)-Pep smart probe, the three signals became apparent at 12 h and gradually intensified with extended incubation time. In contrast, these fluorescence signals remained negligible in cells treated with the control probe PDA-MB-Pep across different time points. The results show that in cells incubated with the smart probe for 12 h, the MB loaded with DOX could specifically bind to the miRNA-34a target, thereby opening the stem–loop structure and releasing a small amount of DOX. The released DOX subsequently activated the p53 pathway, which led to the upregulation of miRNA-34a expression. This upregulation resulted in the opening of additional MB stem–loop structures, releasing more DOX, and thereby initiating a cyclical process involving the probe response’s to miRNA-34a, DOX release, pathway activation, and further upregulation of miRNA-34a. Ultimately, this cyclic process progressively induced cell apoptosis, as evidenced by the increased expression of Caspase-3. In contrast, the control probe, which lacked embedded DOX, was unable to initiate this cycle. These findings indicate that the PDA-MB (DOX)-Pep smart probe could be employed to visually study the effects of drug release on the p53 pathway and pathway-related molecules in cancer cells.

**Figure 6:**
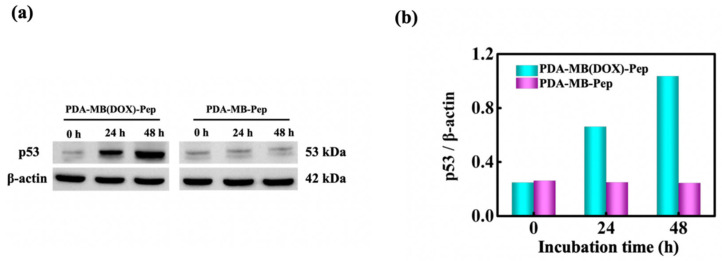
(**a**) WB analysis of p53 in HeLa cells treated with PDA-MB (DOX)-Pep probe and PDA-MB-Pep probe for different durations (0 h, 24 h, 48 h); β-actin served as loading control. (**b**) Quantified band values were normalized to corresponding β-actin signal.

Furthermore, WB analysis was also performed to investigate the activation of the p53 protein after incubation of cells with the probes. Figure 6a,b showed that in cells treated with PDA-MB (DOX)-Pep, the expression of the p53 protein gradually intensified as the incubation time increased. However, in cells treated with the control probe, the p53 protein levels were lower and did not increase with incubation time. These results further demonstrate that the p53 pathway could be activated by the DOX embedded in the PDA-MB (DOX)-Pep nanoprobe.

Finally, to identify cell apoptosis induced by the PDA-MB (DOX)-Pep nanoprobe, an apoptosis detection kit was used. Figure S10 indicates that apoptosis only appeared in cells treated with PDA-MB (DOX)-Pep, confirmed by a red fluorescent membrane and green fluorescent nucleus. However, the control cells exhibited no fluorescence signals, indicating that the PDA-MB (DOX)-Pep probe can induce cell apoptosis. We further evaluated cell apoptosis using Calcein AM/PI double-staining, where PI stained dead cells red and Calcein-AM stained live cells green (Figure S11). The green fluorescence intensities decreased gradually, and conversely, the red fluorescence intensities were gradually enhanced over time. These results imply that the designed PDA-MB (DOX)-Pep nanoprobe can indeed promote apoptosis.

Similarly, after the HepG-2 cells had been pretreated under identical conditions to those used for the HeLa cells, the experimental results of CLSM, WB, and apoptosis detection were consistent with the results obtained for the HeLa cells (Figures S12–S16). All the results showed that upon binding to cellular miRNA-34a, the PDA-MB (DOX)-Pep probe could specifically release DOX and activate the p53 pathway, which induced miR-34a expression and subsequently contributed to cell apoptosis. Therefore, the designed PDA-MB (DOX)-Pep nanoprobe can be used to visually investigate drug activation of the p53 pathway and its impact on miRNA and apoptosis.

### 3.6. Cellular Uptake of the Nanoprobe

The cellular internalization of PDA-MB (DOX)-Pep was also investigated by applying different endocytosis inhibitors [49,50]. The imaging results showed that compared to the control group, the changes in the three fluorescence signals of for the nanoprobe were negligible after cells were pretreated with CPZ and EIPA, suggesting that the endocytosis of the PDA-MB (DOX)-Pep probe is not mediated by clathrin or macropinocytosis. However, the three fluorescence signals in Nystatin-treated cells remarkably decreased, implying that cellular uptake of PDA-MB (DOX)-Pep depends on caveolae-mediated endocytosis (Figures S17 and S18).

## 4. Conclusions

In conclusion, we have developed an innovative fluorescent nanoprobe capable of visually monitoring the impact of cyclical DOX release, which is triggered by miRNA, on the p53 pathway and Caspase-3. The nanoprobe was constructed using PDA NPs modified with a dense shell of MB (DOX) and peptide, which responded to miRNA-34a and Caspase-3 targets, respectively. The nanoprobe was remarkably sensitive and specific to the two targets, and the DOX embedded in the MB was effectively released in a miRNA-34a-responsive manner. Moreover, the results of cell experiments indicated that compared to the control probe, the designed nanoprobe was able to activate the p53 pathway by triggering DOX release, which subsequently upregulated miRNA-34a expression. This initiated a cyclical process of DOX release, p53 activation, and miRNA-34a upregulation, ultimately leading to cell apoptosis. We anticipate that the nanoprobe will offer novel opportunities to visually elucidate the impact of drugs on signaling pathways and related biomolecules at the cellular level, thereby enhancing the efficacy of anticancer therapeutics. Furthermore, the design strategy can provide strong support for the development of combination therapy based on chemotherapy and gene therapy, as well as the screening and efficacy evaluation of new drugs.

## Data Availability

The original contributions presented in this study are included in the article/Supplementary Materials. Further inquiries can be directed to the corresponding authors.

## Supplementary Materials

The following supporting information can be downloaded at https://www.mdpi.com/article/10.3390/bios15060383/s1: Figure S1: Fluorescence spectra of DOX as a function of MB concentration (a); fluorescence intensity quantization of DOX at 560 nm emission wavelength (b); UV-visible absorption spectra of DOX, MB, and MB(DOX) (c). Figure S2: FTIR spectrum of PDA NPs. Figure S3: SEM images of PDA NPs (a) and (b) nanoprobe (scale bar: 100 nm); EDS images of PDA NPs (c) and nanoprobe (d). Figure S4: UV-vis spectra of MB (DOX), peptide, PDA NPs, and nanoprobe. Figure S5: Fluorescence quenching spectra and fluorescence intensity profiles. Figure S6: Specificity of nanoprobe against miRNA-34a (a), Caspase-3 (b), and other interfering substances. Figure S7: Kinetic analysis of nanoprobe. Figure S8: Fluorescence intensity changes over time of DOX, Texas Red, and Cy5 at different pH values (a) and in cell culture medium (b). Figure S9: Survival rates of HeLa (a) and HepG-2 cells (b) treated with PDA-MB-Pep and PDA-MB (DOX)-Pep for different durations. Figure S10: CLSM images of HeLa cells stained with apoptosis detection kit. Figure S11: CLSM images of HeLa cells stained with Calcein-AM/PI kit. Figure S12: CLSM images of HepG-2 cells cultured with 7 μg/mL PDA-MB (DOX)-Pep nanoprobe for different durations. Figure S13: CLSM images of HepG-2 cells cultured with 7 μg/mL PDA-MB-Pep nanoprobe for different durations. Figure S14: Western blot analysis of p53 in HepG-2 cells treated with PDA-MB (DOX)-Pep probes and PDA-MB-Pep probes for different durations. Figure S15: CLSM images of HepG-2 cells stained with apoptosis detection kit. Figure S16: CLSM images of HepG-2 cells stained with Calcein-AM/PI kit. Figure S17: Cellular uptake pathway study in HeLa cells. Figure S18: Cellular uptake pathway study in HepG-2 cells. Table S1: The sequences of the DNA and peptide chains used in this study.
