# Supplementary material for: A Smart Nanoprobe for Visually Investigating the Activation Effect of Cyclical DOX Release on the p53 Pathway and Pathway-Related Molecules
Source: Biosensors (Basel). 2025 Jun 13;15(6):383. doi: 10.3390/bios15060383 (PMC12191000; doi:10.3390/bios15060383)
Supplement: Supplementary file 1 [file biosensors-15-00383-s001.zip › biosensors-3657485-supplementary.pdf]

biosensors

MDPI

*Supplementary Materials:*

# A Smart Nanoprobe for Visually Investigating the Activation Effect of Cyclical DOX Release on the p53 Pathway and Pathway-Related Molecules

**Ping Sun [1,†], Chunlei Gao [1,†], Zhe Chen [1], Siyu Wang [1], Gang Li [2], Mingming Luan [1,*] and Yaoguang Wang [1,*]**

**Experimental Details:**

*Materials.* Dopamine hydrochloride was purchased from Solarbio company (Beijing, China); Doxorubicin hydrochloride was obtained from Saan Chemical Technology (Shanghai, China) Co., Ltd; Tris (hydroxymethyl) methyl aminomethane, dimethyl sulfoxide (DMSO), $CaCl_2$, NaCl, $MgCl_2$ and ethanol were purchased from Chemical Reagent company (Shanghai, China); Caspase-3 was purchased from Abcam (Shanghai, China); 3-(4,5-dimethylthiazol-2-yl)-2,5-diphenyltetrazolium bromide (MTT) was purchased from Sigma-Aldrich (Louis, Missouri, USA); Fetal bovine serum, DMEM, cyan chain double antibody and trypsin were purchased from Biological Industries (Shanghai, China); Glutathione, hemoglobin and bovine serum albumin were purchased from Sangon Biotech company (Shanghai, China); Phosphate buffer solution (PBS), A549 (human lung cancer cell line), HepG-2 (human liver cancer cell line), HeLa (human cervical cancer cell line) and MCF-7 (human breast cancer cell line) were purchased from Punosai Life Technology company (Wuhan, China); A549 (human lung cancer cell line), phosphate buffer solution (PBS), HepG-2 (human liver cancer cell line), HeLa (human cervical cancer cell line) and MCF-7 (human breast cancer cell line) were purchased from Punosai Life Technology Company (Wuhan, China). Caspase-3 activity and Annexin V apoptosis were purchased from Biyuntian (Shanghai, China). This experiment used only pure water from Wahaha Group (Hangzhou, China).

*Instruments.*UV-vis spectrometer (UV 1920, Lengguang Technology Co., Ltd. Shanghai, China); Fluorescence spectrometer (F97 Pro, Lengguang Technology Co., Ltd., Shanghai, China); Microplate reader (Multiskan FC, Thermo Scientific); pH meter (Shanghai Leici Instrument Factory); Transmission electron microscopy (JEM 2100, Japan); Scanning electron microscopy (Regulus 8220, Japan); Fourier transform infrared (FT-IR) spectrometer (Nicolet10, USA).

*Software:* Origin 9.7; Image J 1.8; LAS AF Lite 1.9.

*Preparation of MB and Peptide*

The oligonucleotides used in this experiment were synthesized by Sangon Biotech company and purified using high-performance liquid chromatography (HPLC). The peptide used in this study was synthesized by Kingsray Biotechnology company and also purified by HPLC. The specific sequences of the DNA and peptide used in this study can be found in Table S1.

*Binding Study of MB and DOX*

DOX (1 μM) was added to different concentrations of MB (0, 50, 100, 150, 200, 300, 400, 500, 600 nM) and incubated for 1 h at room temperature to gain MB (DOX). DOX

 https://doi.org/10.3390/bios15060383

was excited at 490 nm and the fluorescent signal was collected in 525-575 nm. The experiments were conducted in three parallel groups under consistent conditions.

## Kinetic Response of Nanoprobe

For exploring the kinetic response of nanoprobe to miRNA-34a and Caspase-3. Perfectly matched miRNA-34a target strand (5 μM) was added to the nanoprobe solution and incubated at 37 °C for different times (0, 10, 20, 30, 40, 60, 100, 120, 150, 180, 240 min). Similarly, Caspase-3 (3 U) was treated with the nanoprobe and incubated for varying times (0, 0.5, 1, 2, 3, 4 h). The fluorescence intensities of Texas Red and Cy5 were measured at λex/λem = 595 nm/615 nm and λex/λem = 625 nm/660 nm, respectively. Three experiments were conducted in parallel.

### Specificity Experiments

To test the specificity of nanoprobe to miRNA-34a and Caspase-3, perfectly matching miRNA-34a target strand (5 μM), Caspase-3 (3 U) and other interfering substances, including other DNA targets (5 μM), hemoglobin (HGB, 6 μg/mL), bovine serum albumin (BSA, 6 μg/mL), glutathione (GSH, 6 μg/mL), and ions (200 mM) were added to the nanoprobe solution for 2 h. The fluorescent signals of Texas Red and Cy5 were measured at appropriate excitation and emission wavelengths. Three groups were measured in parallel.

### Stability of Nanoprobe

For assessing the stability of the nanoprobe, the three fluorescence signals of DOX, Texas Red and Cy5 were investigated after being incubated in different pH (2-10) buffers and in cell culture medium for various storage times (0-72 h). The, fluorescence intensities were collected (DOX: λex = 490 nm, λem = 560 nm; Texas Red: λex = 595 nm, λem = 615 nm; Cy5: λex = 625 nm, λem = 660 nm).

### Cell Culture

All cells used in the experiments were cultured in DMEM (1% antibiotics penicillin/streptomycin and 10% fetal bovine serum) and incubated at 37 °C in a 5% $CO_2$.

### MTT Assay

To detect the biological toxicity of PDA NPs and PDA-MB(DOX)-Pep nanoprobe, MTT assays were used to determine the cell viability. HeLa and HepG-2 cells were inoculated in 96 wells ($10^6$ cells/well) and incubated overnight. After culture medium was removed, and PDA NPs (70 μg/mL) and nanoprobe solution (7 μg/mL) were added. After incubation for different times (0, 6, 12, 24, 48, 72 h), MTT (5 mg/mL, 10 μL) was added and incubated for 4 hours. Finally, the supernatant was discarded, and DMSO (150 μL) was added. The absorbance at a wavelength of 490 nm was measured on a Multiskan FC. Cell viability was calculated based on the absorbance normalized to the data from untreated cells.

### Western Blot

After incubation with the nanoprobe for different times (0 h, 24 h, 48 h), respectively, the cells were lysed and cellular p53 protein expression was detected by Western Blotting (WB). Firstly, the cells were washed twice with PBS buffer (pre-cooling), and then the lysate (150 μL) was added. The concentration of p53 protein was determined by BCA protein kit. After the sample to be tested was mixed with SDS-PAGE buffer (5X)

and boiled, the sample was separated by electrophoresis and the gel was transferred to PVDF. After the membrane was transferred, it was placed on a shaker, washed with TBST (5-10 min/time, 3 times), and sealed at room temperature. After the blocking solution was removed, the primary antibody was added, and the membrane was washed with TBS-T (10 min/time, 3 times) after overnight on the shaker. The secondary antibody was added and incubated at room temperature for 2 h. After the second antibody incubation, the membrane was washed with TBST (5-10 min/time, 3 times). Finally, the enhanced chemiluminescence signal was collected by gel imager using ECL.

*Cellular Uptake of Nanoprobe*

To explore the cellular uptake of fluorescent nanoprobe, Hela and HepG-2 cells were divided into four groups in parallel and pre-treated with PBS buffer, chlorpromazine (CPZ), EIPA and nystatin (Nystatin) for 2 h respectively. After incubation with 7 μg/mL nanoprobe for 24 h, the CLSM imaging was performed.

**Table S1**. The sequences of the DNA and peptide chains used in this study

| Oligonucleotide | Sequences (from 5′ to 3′) |
|---|---|
| MB | 5′-Texas Red CGTGCACAACCAGCTA AGACACTGCACG-3′ |
| miRNA-34a | 5′-GCAGTGTCTTAGCTGGTTGT-3′ |
| mismiRNA-34a | 5′-GCAGTGTCTTATCTGGTTGT-3′ |
| peptide | Cy5-Ahx-Gly-Gly-Asp-Glu-Val-Asp-Gly-Gly-Cys |
| miRNA-67 | 5′-TCTGCACACCTCTTGACACTCCG-3′ |
| miRNA-21 | 5′-TAGCTTATCAGACTGATGTTGA-3′ |
| miRNA-221 | 5′-AGCTACATTGTCTGCTGGGTTTC-3′ |

3.1. The interact

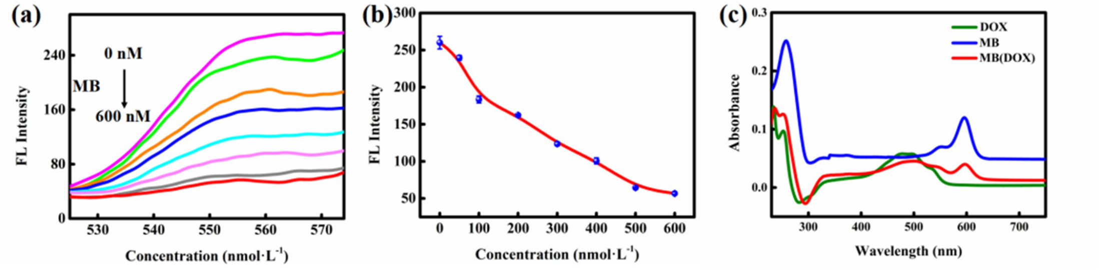


**Figure S1.** (a) Fluorescence spectra of DOX as a function of the concentrations of MB. (b) Fluorescence intensity quantization of DOX at 560 nm emission wavelength; (c) UV-visible absorption spectra of DOX, MB and MB(DOX).

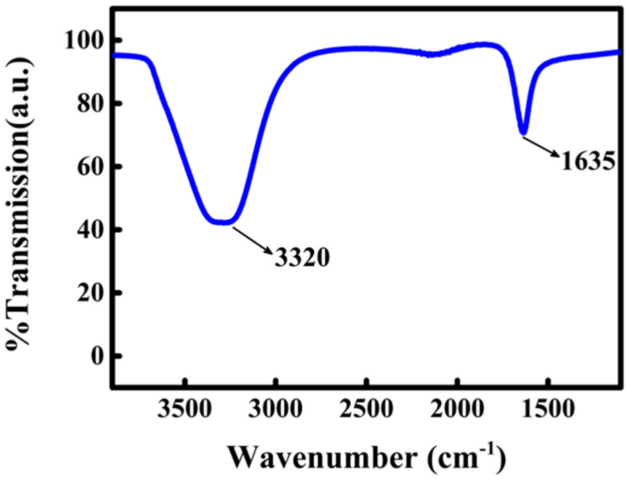


**Figure S2.** FTIR spectrum of PDA NPs.

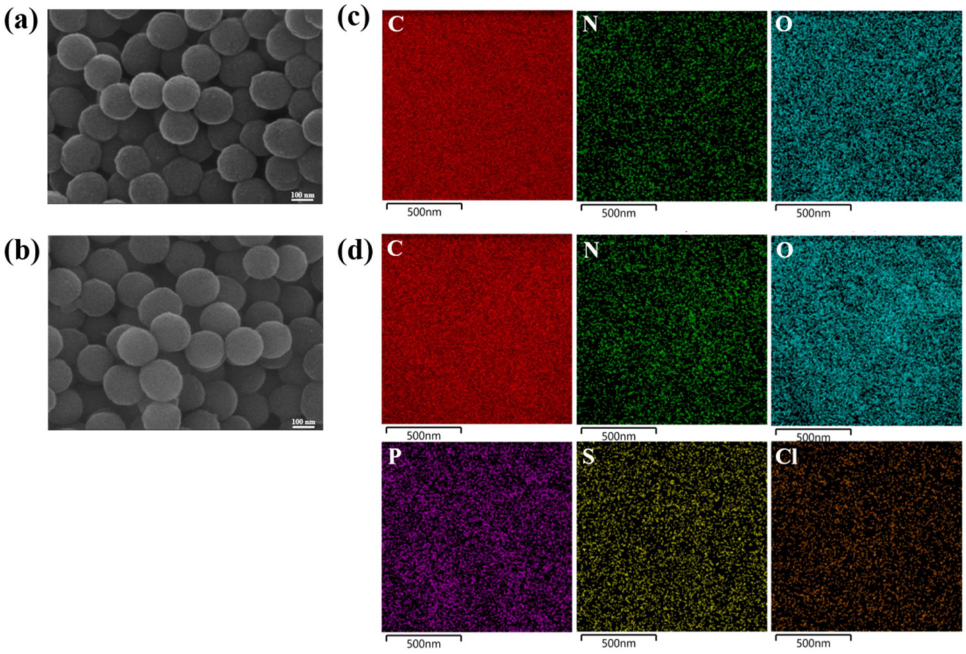


**Figure S3.** SEM images of PDA NPs (a) and (b) the nanoprobe (Scale bar: 100nm). EDS images of PDA NPs (c) and the nanoprobe (d).

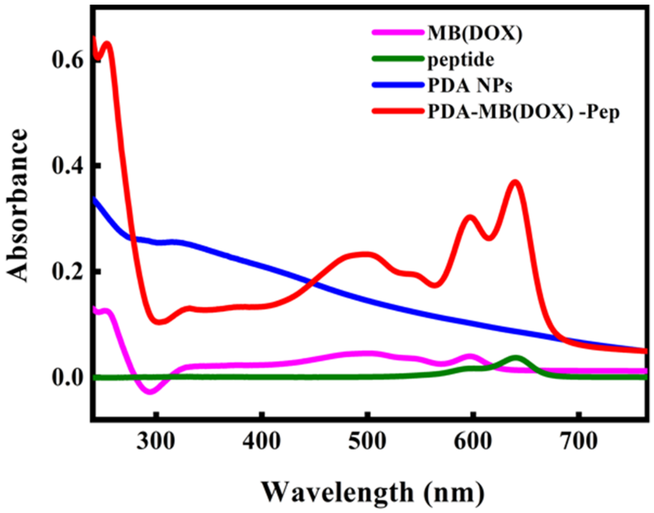


**Figure S4.** UV-vis spectra of MB (DOX), peptide, PDA NPs and the nanoprobe.

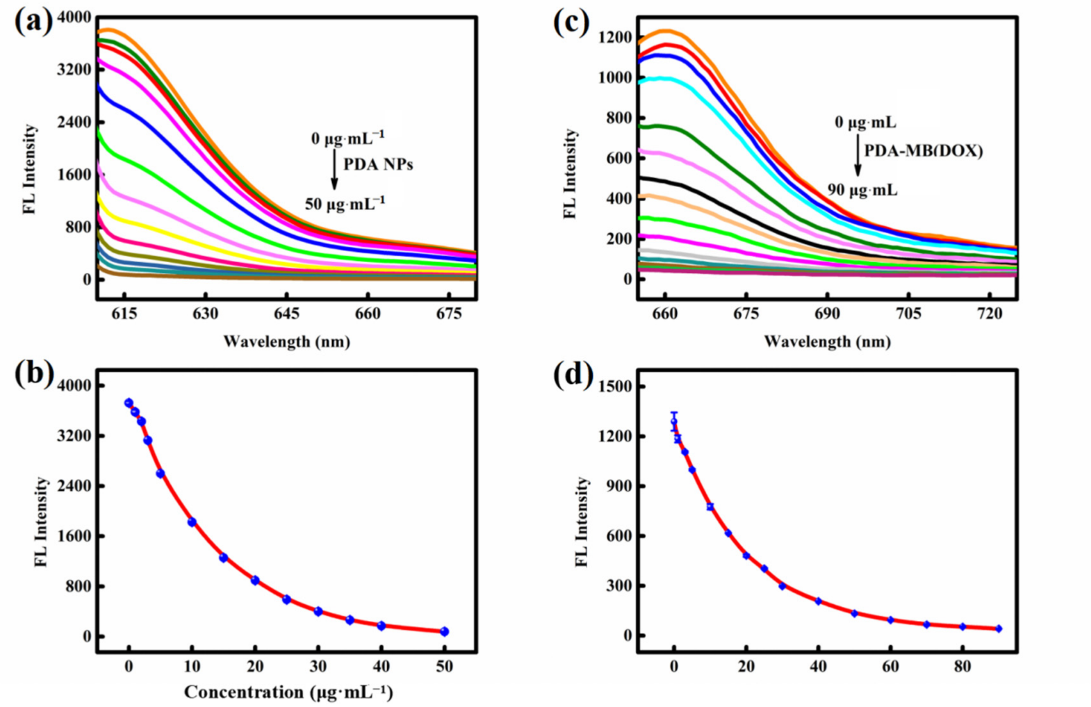


**Figure S5.** Fluorescence quenching spectras (a) and fluorescence intensity profiles at 615 nm (b) of Texas Red-MB treated with different concentrations of PDA NPs. The fluorescence quenching spectras (c) and fluorescence intensity profiles at 670 nm (d) of Cy5-peptidetreated with different concentrations of PDA-MB(DOX).

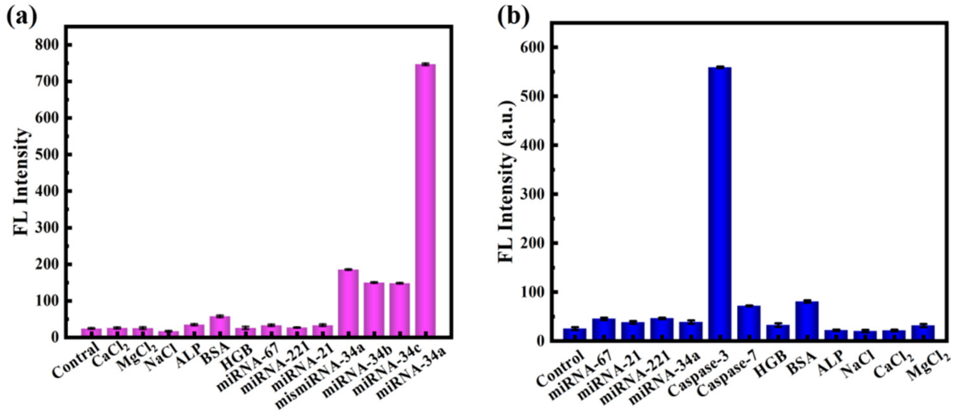


**Figure S6.** (a) Specificity of the nanoprobe against miRNA-34a and other interfering substances. (b) Specificity of the nanoprobe against Caspase-3 and other interfering substances.

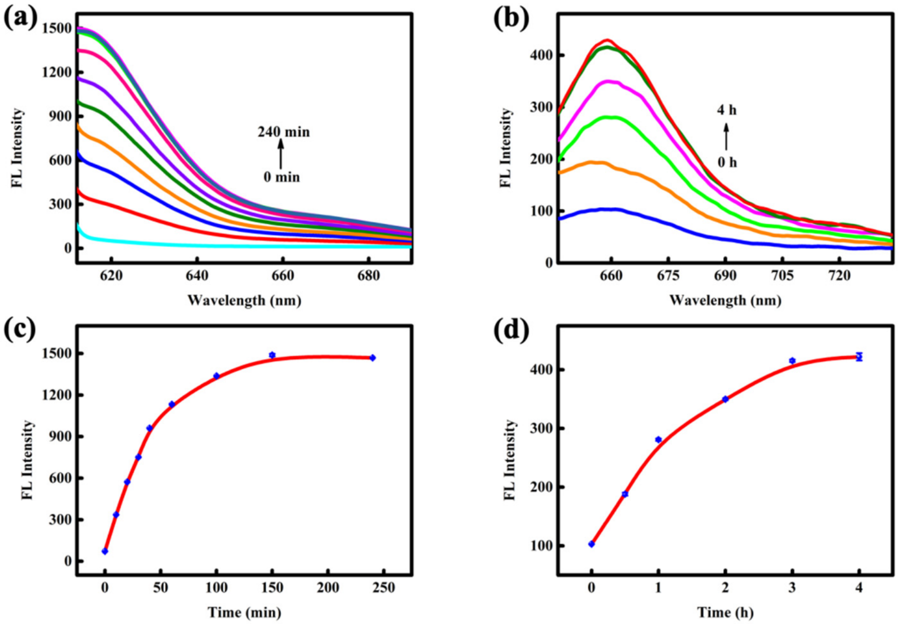


**Figure S7.** Kinetic analysis of nanoprobe in the presence of miRNA-34a (a) and Caspase-3 (b). Fluorescence intensity plots for Texas Red at 615nm (c) and Cy5 at 660nm (d).

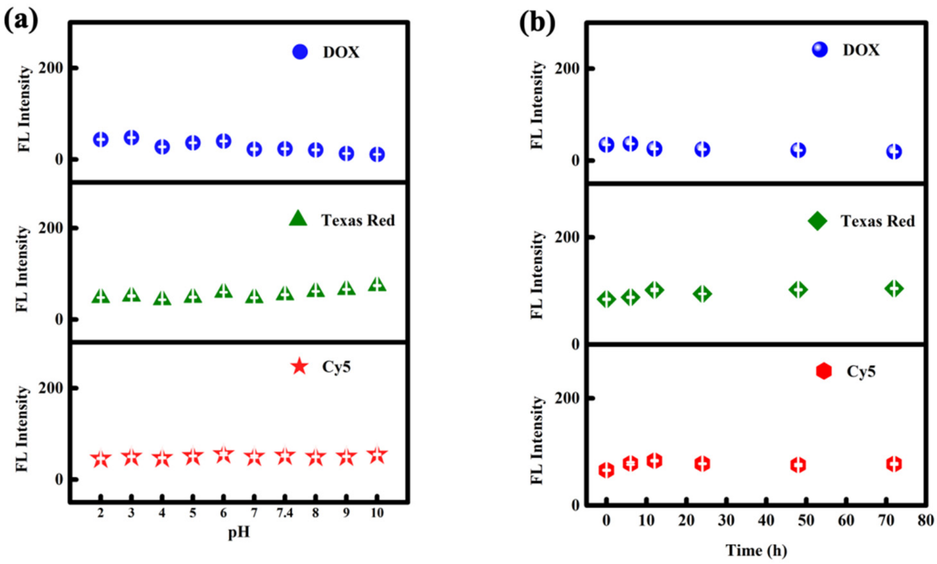


**Figure S8.** Fluorescence intensity changes of DOX, Texas Red and Cy5 with different pH values (a) and in cell culture medium over time (b).

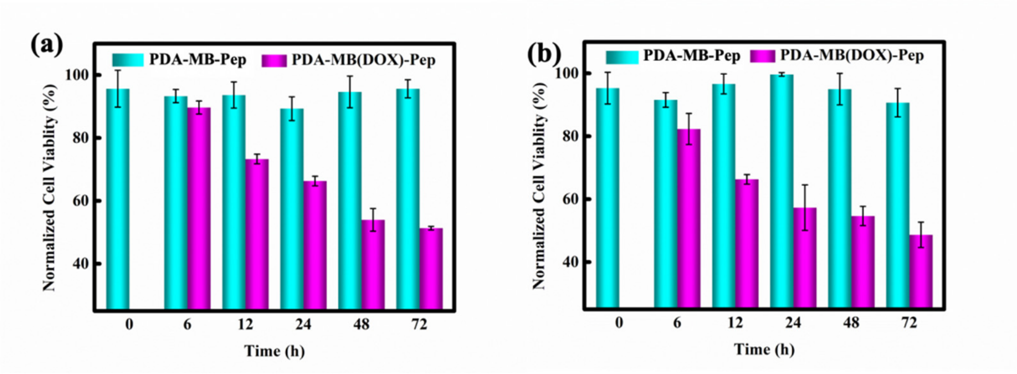


**Figure S9.** Survival rate of HeLa (a) and HepG-2 cells (b) treated with PDA-MB-Pep and PDA-MB(DOX)-Pep for different times.

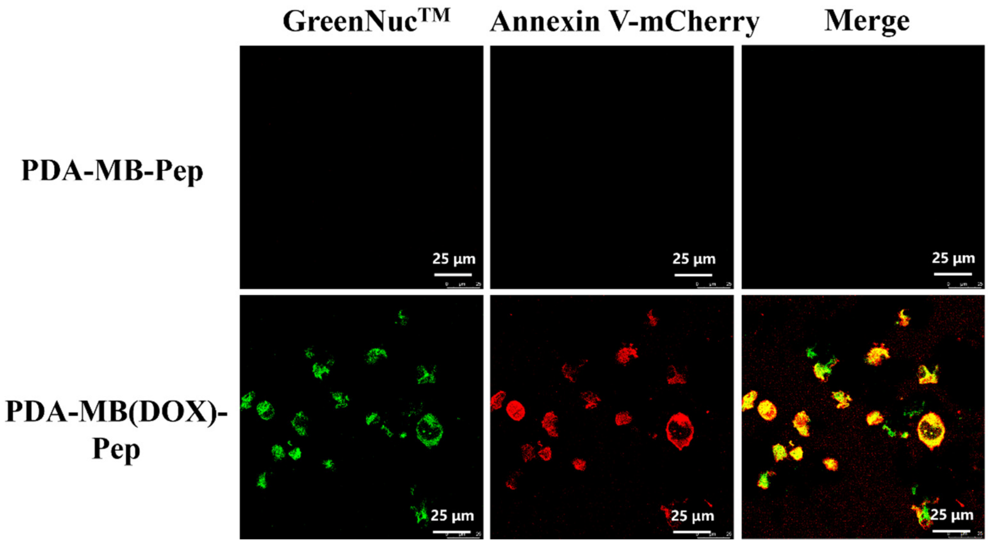

**Figure S10.** CLSM images of HeLa cells stained with the apoptosis detection kit. HeLa cells were pretreated with PDA-MB(DOX)-Pep and PDA-MB-Pep (control) for 48 h. Scale bars: 25 µm.

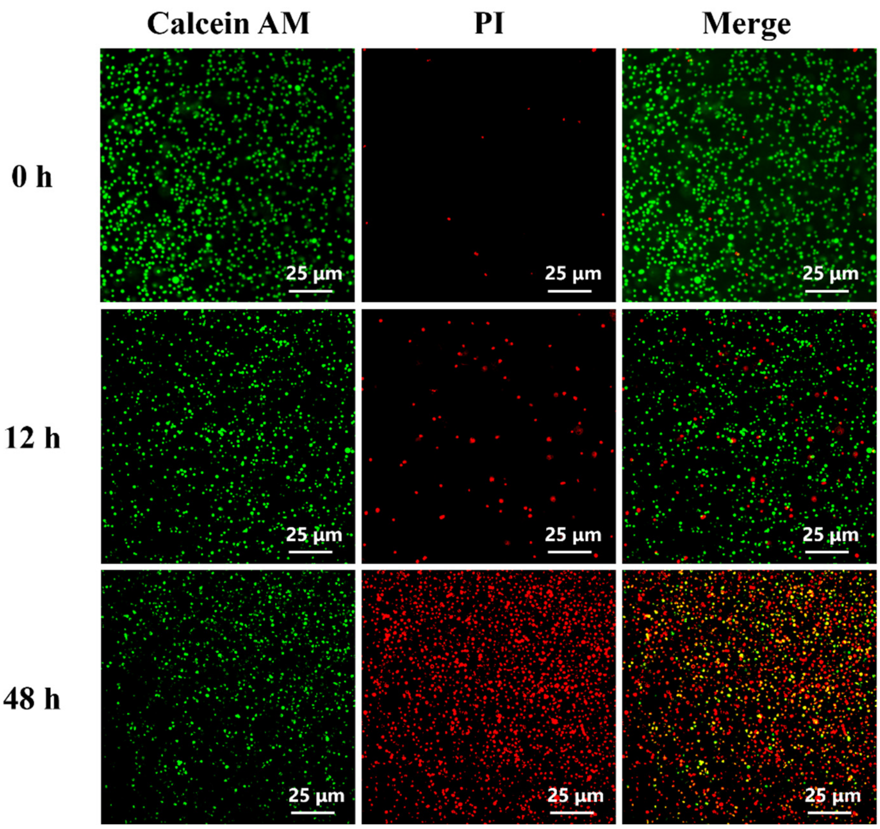


**Figure S11.** CLSM images of HeLa cells stained with Calcein-AM/PI kit. Cells were pretreated with PDA-MB(DOX)-Pep for different times. Scale bars: 25 µm.

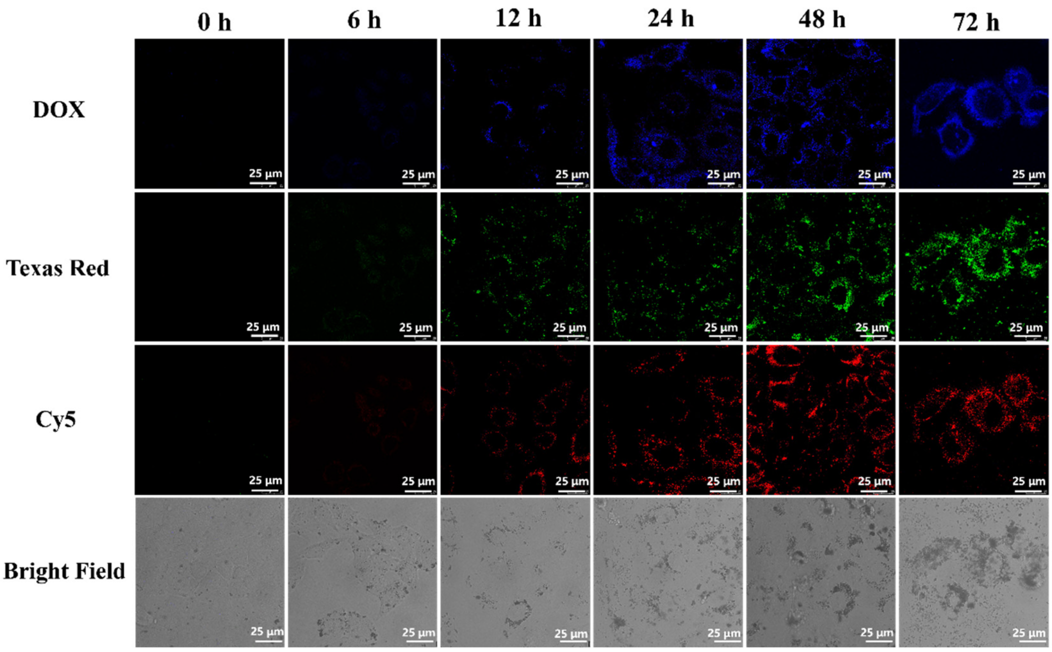

**Figure S12.** CLSM images of HepG-2 cells cultured with 7 μg/mL of PDA-MB(DOX)-Pep nanoprobe for different times. The DOX (blue), Texas Red (green) and Cy5 (red) channels were excited at 490, 595 and 625 nm, respectively. Scale bars: 25 μm.

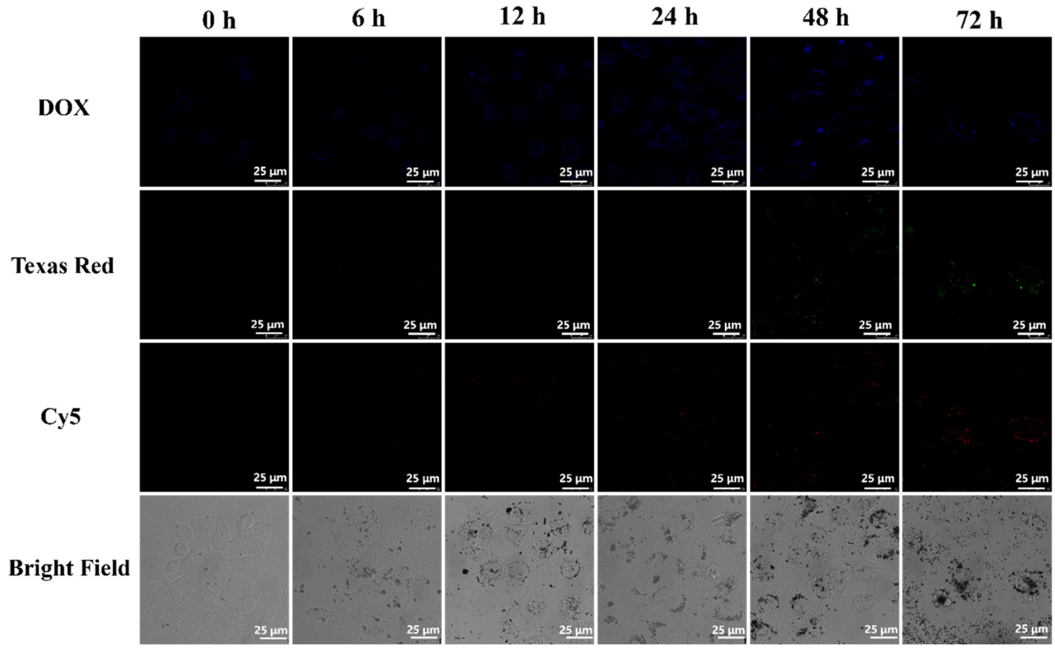


**Figure S13.** CLSM images of HepG-2 cells cultured with 7 μg/mL PDA-MB-Pep nanoprobe for different times. The DOX (blue), Texas Red (green) and Cy5 (red) channels were excited at 490, 595 and 625 nm, respectively. Scale bars: 25 μm.

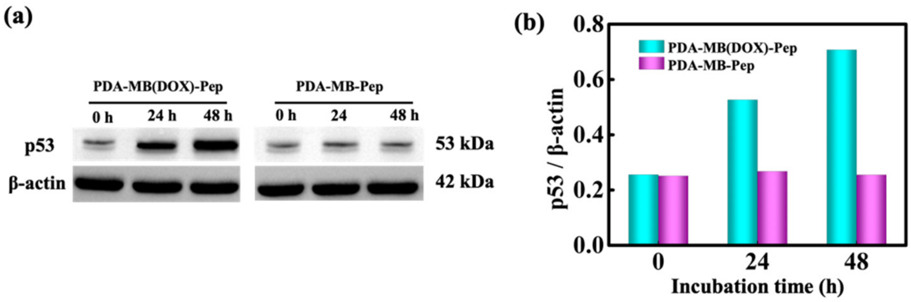


**Figure S14.** Western blot analysis of p53 in HepG-2 cells treated with PDA-MB(DOX)-Pep probes and PDA-MB-Pep probes for different times (0 h, 24 h, 48 h), β-actin served as the loading control.

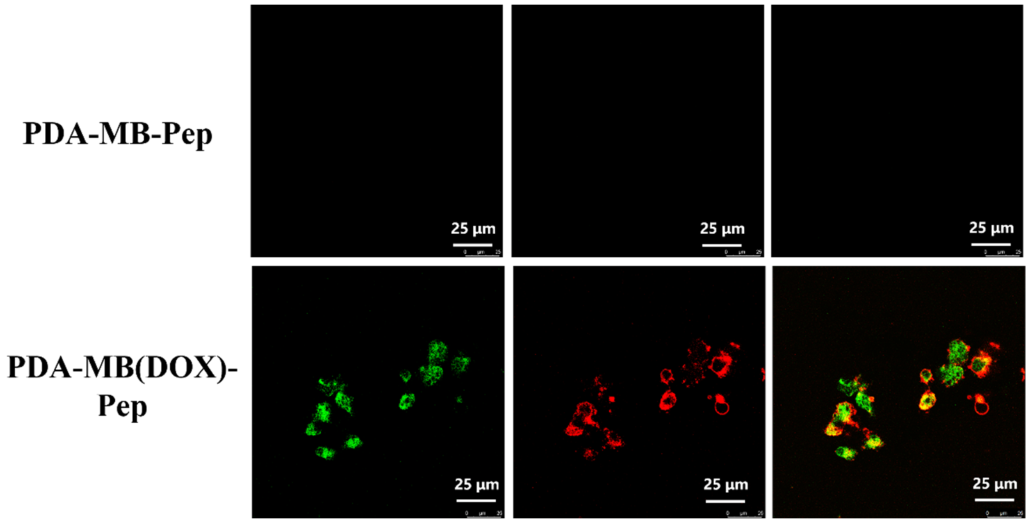


**Figure S15.** CLSM images of HepG-2 cells stained with the apoptosis detection kit. HepG-2 cells were pretreated with PDA-MB(DOX)-Pep and PDA-MB-Pep (control) for 48 h. Scale bars: 25 μm.

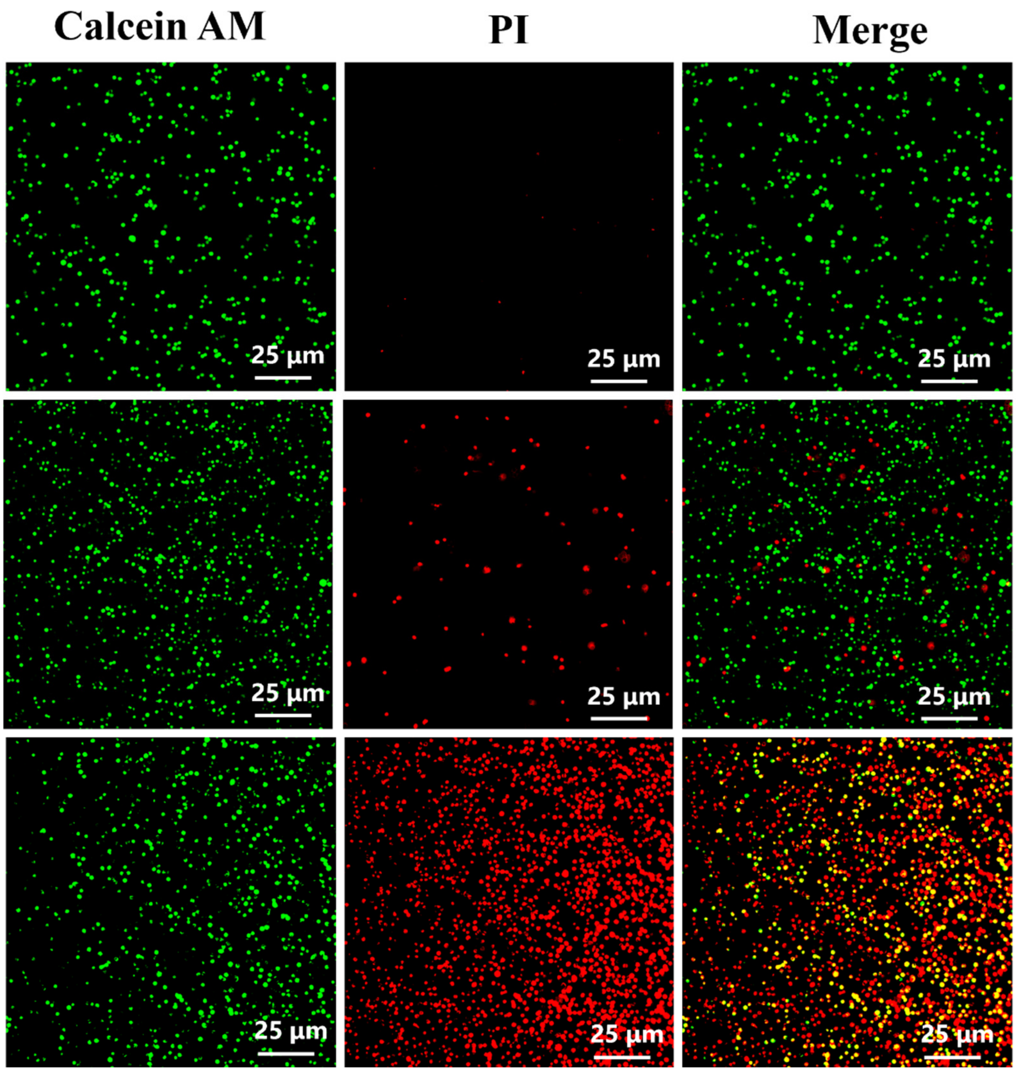


**Figure S16.** CLSM images of HepG-2 cells stained with Calcein-AM/PI kit. Cells were pretreated with PDA-MB(DOX)-Pep for different times. Scale bars: 25 μm.

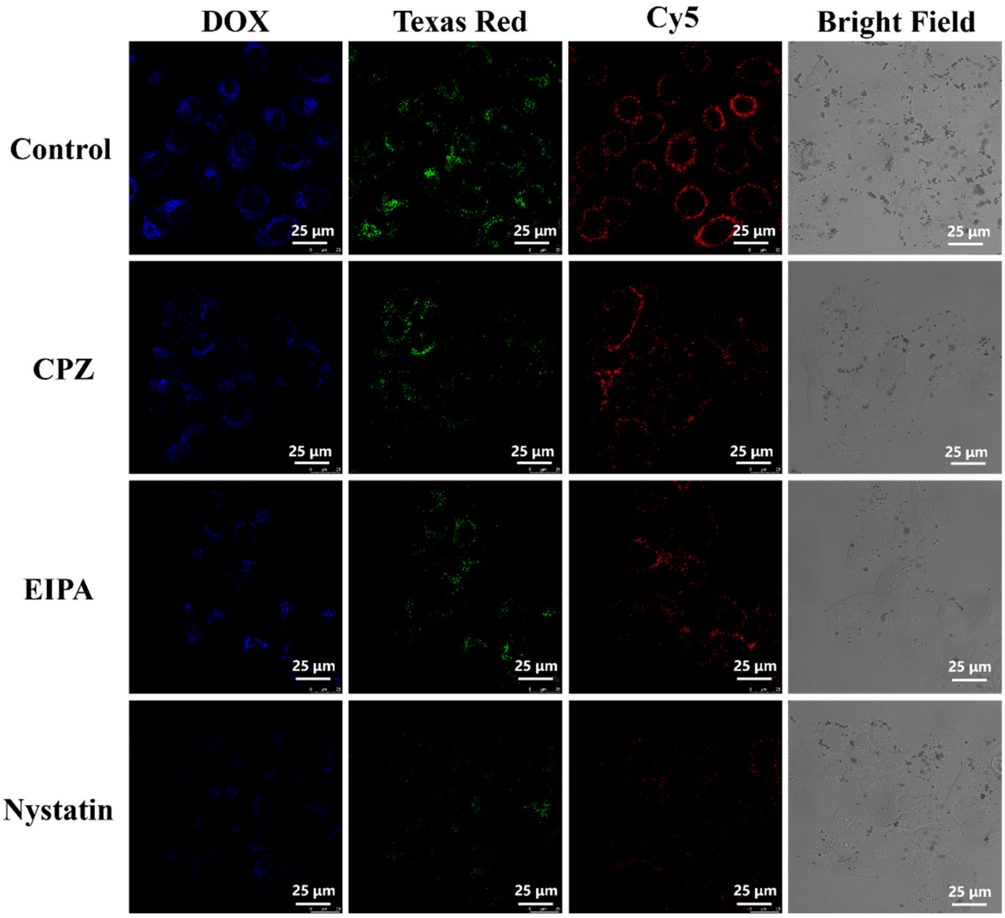


**Figure S17.** Cell uptake pathway study. HeLa cells were treated with CPZ, EIPA and Nystatin before incubated with 7 μg/mL PDA-MB(DOX)-Pep. Cells without treatment served as a control. DOX (blue), Texas Red (green) and Cy5 (red) channels were excited at 490, 595 and 625 nm, respectively. Scale bars: 25 μm.

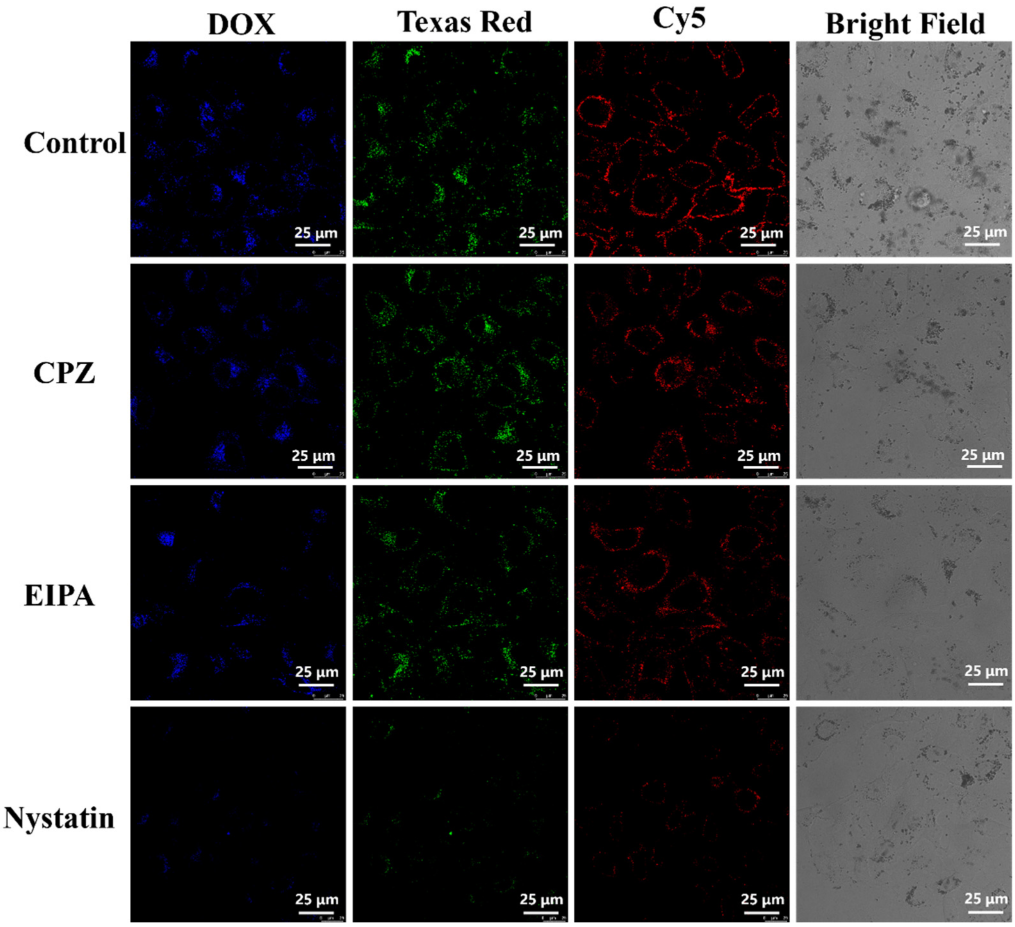


**Figure S18.** Cell uptake pathway study. HepG-2 cells were treated with CPZ, EIPA and Nystatin before incubated with 7 μg/mL PDA-MB(DOX)-Pep. Cells without treatment served as a control. DOX (blue), Texas Red (green) and Cy5 (red) channels were excited at 490, 595 and 625 nm, respectively. Scale bars: 25 μm.

**Disclaimer/Publisher's Note:** The statements, opinions and data contained in all publications are solely those of the individual author(s) and contributor(s) and not of MDPI and/or the editor(s). MDPI and/or the editor(s) disclaim responsibility for any injury to people or property resulting from any ideas, methods, instructions or products referred to in the content.
